# Long noncoding RNA DGCR5 involves in tumorigenesis of esophageal squamous cell carcinoma via SRSF1-mediated alternative splicing of Mcl-1

**DOI:** 10.1038/s41419-021-03858-7

**Published:** 2021-06-07

**Authors:** Yuqing Duan, Yunlong Jia, Jiali Wang, Tianxu Liu, Zishuo Cheng, Meixiang Sang, Wei lv, Jing Qin, Lihua Liu

**Affiliations:** 1 Department of Tumor Immunotherapy, Fourth Hospital of Hebei Medical University, 050035 Shijiazhuang, Hebei China; 2 Department of Research Center, Fourth Hospital of Hebei Medical University, 050035 Shijiazhuang, Hebei China; 3 Hebei Cancer Institute, 050011 Shijiazhuang, Hebei China; 4 Department of General Surgery, Shijiazhuang Third People’s Hospital, 050011 Shijiazhuang, Hebei China; 5 International Cooperation Laboratory of Stem Cell Research, Hebei Medical University, 050011 Shijiazhuang, Hebei China

**Keywords:** Tumour biomarkers, Apoptosis

## Abstract

Long noncoding RNAs (lncRNAs) emerge as essential roles in the regulation of alternative splicing (AS) in various malignancies. Serine- and arginine-rich splicing factor 1 (SRSF1)-mediated AS events are the most important molecular hallmarks in cancer. Nevertheless, the biological mechanism underlying tumorigenesis of lncRNAs correlated with SRSF1 in esophageal squamous cell carcinoma (ESCC) remains elusive. In this study, we found that lncRNA DiGeorge syndrome critical region gene 5 (DGCR5) was upregulated in ESCC clinical samples, which associated with poor prognosis. Through RNA interference and overexpression approaches, we confirmed that DGCR5 contributed to promote ESCC cell proliferation, migration, and invasion while inhibited apoptosis in vitro. Mechanistically, DGCR5 could directly bind with SRSF1 to increase its stability and thus stimulate alternative splicing events. Furthermore, we clarified that SRSF1 regulated the aberrant splicing of myeloid cell leukemia-1 (Mcl-1) and initiated a significant Mcl-1L (antiapoptotic) isoform switch, which contributed to the expression of the full length of Mcl-1. Moreover, the cell-derived xenograft (CDX) model was validated that DGCR5 could facilitate the tumorigenesis of ESCC in vivo. Collectively, our findings identified that the key biological role of lncRNA DGCR5 in alternative splicing regulation and emphasized DGCR5 as a potential biomarker and therapeutic target for ESCC.

## Introduction

Esophageal cancer (EC) is one of the most aggressive cancer worldwide and contributes the sixth highest cancer-related mortality rate^1^. Disease carcinogenesis and prevention substantially distinctive between esophageal adenocarcinoma (EAC) and esophageal squamous cell carcinoma (ESCC), with obvious differences in histological types^2^. ESCC accounts for up to 90% of esophageal cancer in the Asian population, which is characterized by high invasion or distant metastasis^3^. Although advances in ESCC treatments approaches such as surgery, chemotherapy, and radiotherapy, the overall 5-year survival rate is still unsatisfied^4^. Therefore, it is urgent to investigate the new biomarkers and treatments for ESCC.

Long noncoding RNAs (lncRNAs) are a class of transcripts longer than 200 nucleotides, most of which lack valid coding capacity^5^. Accumulating studies have reported lncRNAs are aberrantly expressed in human cancers and participate critically roles in diverse biological processes^6,7^. Mechanistically, lncRNAs function as important regulators in gene expression networks in multiple ways, such as chromatin modification, transcription, and posttranscriptional regulation, which mainly depend on their subcellular localization^8^. For example, HNF1A-AS1 in gastric cancer^9^ and TTN-AS1 in non-small cell lung cancer (NSCLC) progression^10^ functioning as the ceRNA molecular is widely reported in cancer development. Besides, lncRNAs regulate their target gene expressions via binding with RNA-binding proteins (RBPs)^11,12^. DiGeorge syndrome critical region gene 5 (DGCR5), also known as linc00037, is a lncRNA located on chromosome22 (22q11.21), which is first reported in Huntington’s disease. Emerging studies indicate that higher expression of DGCR5 is particularly important in the progression of laryngeal carcinoma^13^ and gallbladder cancer^14^, suggesting DGCR5 may be an oncogenic lncRNA in cancers. By contrast, DGCR5 acts as a tumor suppressor in cervical cancer^15^, gastric cancer^16^, and bladder cancer^17^. All these seemingly contradictory findings prove the heterogeneity roles of DGCR5 in different biological scenarios. As for ESCC, research works have confirmed that some aberrantly expressed lncRNAs related to ESCC development^18,19^. However, the functions and mechanisms of DGCR5 in ESCC remain unknown.

Alternative splicing (AS) is a posttranscriptional process enabling to regulate the generation of various mRNA and protein products, playing a vital role in the process of development and differentiation^20^. Importantly, aberrant AS events are frequently observed in a diverse spectrum of tumor types, which is independent of the already characterized genetic alterations^21^. Serine- and arginine-rich splicing factor 1 (SRSF1) belongs to the splicing factor of the SR family, and participates in posttranscriptional gene regulation through mRNA alternative splicing^22^. Our previous research has demonstrated that SRSF1 could mediate the aberrant AS of bridging integrator-1 (BIN1) to neutralize its tumor-suppressing functions in NSCLC^23^. Nevertheless, the concrete function and upstream regulatory mechanisms of SRSF1 in ESCC still remain elusive. Recently, the interactions between lncRNAs and splicing factors are regarded to be important for initiating and maintaining the alternative splicing process^24^. Nevertheless, the potential function of DGCR5 on ESCC and whether it relates to the AS events via SRSF1-regulated have never been discovered.

The aim of this study is to characterize the biological functions of DGCR5 in ESCC and investigate the underlying mechanisms associated with alternative splicing. In this study, we first identified an upregulated expression of DGCR5 in ESCC. Gain-of-function and loss-of-function experiments investigated the carcinogenesis of DGCR5 on ESCC cells in vitro and in vivo. Then, the interaction between DGCR5 and SRSF1 was analyzed to explore the impact of DGCR5 on the alternative splicing process of myeloid cell leukemia-1 (Mcl-1). Taken above, our results shed new light on the role of DGCR5 in the tumorigenesis and progression of ESCC.

## Results

### DGCR5 is upregulated in human ESCC tissues and DGCR5 overexpression correlates with poor prognosis

**Fig. 1:**
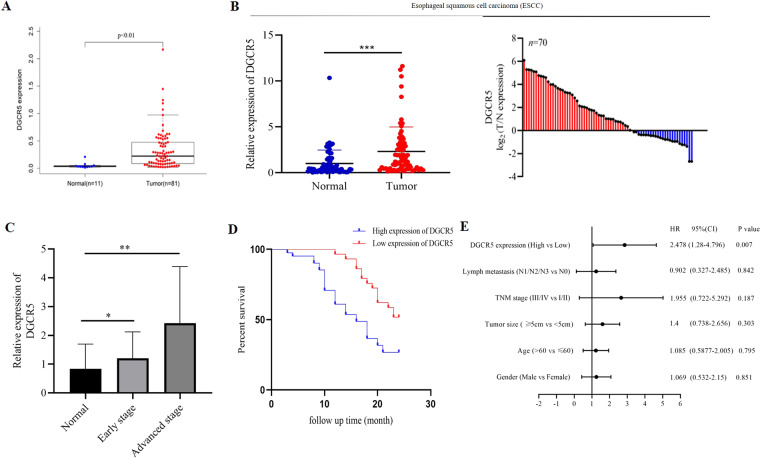
The expression of DGCR5 in human ESCC tissues and normal tissue samples. **A** DGCR5 expression level was observed in ESCC tissues from the TCGA database. **B** DGCR5 expression was analyzed by qRT-PCR from 70 pairs of ESCC samples and non-tumor tissues. GAPDH was used as a control. **C** DGCR5 expression levels in different tumor stages. **D** Kaplan–Meier survival analysis showed the relationship between DGCR5 and prognosis. **E** DGCR5 is an independent prognostic factor in ESCC patients. **P < 0.01 and *P < 0.05.

To investigate the function of lncRNA DGCR5 in ESCC tissues, we first analyzed DGCR5 expression from the TCGA database (https://cancergenome.nih.gov/) and found that DGCR5 was significantly upregulated in ESCC tissues compared with normal tissues (Fig. 1A). Next, we detected the level of DGCR5 in another 70 paired samples of ESCC and matched adjacent normal tissues by qRT-PCR, in which DGCR5 expression was significantly elevated in 58.57% (41 of 70) of ESCC tissues (Fig. 1B). Furthermore, we examined the expression of DGCR5 was positively associated with the TNM stage of ESCC, suggesting that higher expression of DGCR5 was significantly correlated with advanced ESCC patients (Fig. 1C). Taken together, these results strongly indicated that DGCR5 contributed to ESCC tumorigenesis as an oncogenic lncRNA.

Aiming at the clinicopathologic significance of DGCR5 in ESCC carcinogenesis, the relationship between DGCR5 expression and multiple clinicopathological parameters of 70 ESCC patients was analyzed. Remarkably, increased DGCR5 expression in ESCC tissues was significantly related to a deeper invasion range (*P* = 0.0252), more lymph node metastasis (*P* = 0.0008) and higher TNM stage (*P* = 0.0008), while not related with age and gender (Table 1). Then, we investigated the connection between DGCR5 expression and the prognosis of ESCC patients. Kaplan–Meier analysis showed that a higher level of DGCR5 was likely to be poor 2-year disease-free survival (DFS) rate for ESCC patients (Fig. 1D), suggesting that elevated DGCR5 was correlated with poor prognosis. Moreover, COX proportional hazard regression analysis was performed to evaluate the prognostic factors for ESCC patients. Covariates included in Cox proportional hazards model are gender, age, TNM stage, lymph node metastasis, invasion range, and DGCR5 expression. Univariate Cox regression analysis showed that TNM stage (*P* = 0.045), lymph node metastasis (*P* = 0.047), and DGCR5 expression (*P* = 0.003) were significantly associated with the prognosis of ESCC patients (Supplementary Table 1). Furthermore, multivariate analysis (Supplementary Table 2) found that DGCR5 expression was considered to be an independent prognostic factor for ESCC patients (*P* = 0.007, Fig. 1E). These results suggested that DGCR5 was upregulated in ESCC tissues and overexpression of DGCR5 could predict a poor clinical prognosis.

**Table 1:** The relationship of lncRNA DGCR5 and clinical characteristics in 70 ESCC patients.

Clinicopathological features	*N*	Expression of DGCR5		χ^2^	*P* value
		High (*n* = 41)	Low (*n* = 29)		
Gender				1.349	0.2452
Male	51	32	19		
Female	19	9	10		
Age (*t*/a)				0.1501	0.6985
≤60	26	16	10		
>60	44	25	19		
Invasion range				5.011	0.0252
T1 + T2	15	5	10		
T3	55	36	19		
TNM stage				11.27	0.0008
I + II	34	13	21		
III+IVA	36	28	8		
Metastasis (lymph)				11.32	0.0008
No	25	8	17		
Yes	45	33	12		

UICC/AJCC TNM classification (8th edition), *P < 0.05, which was considered as a significant difference.

### DGCR5 promotes ESCC progression in vitro

**Fig. 2:**
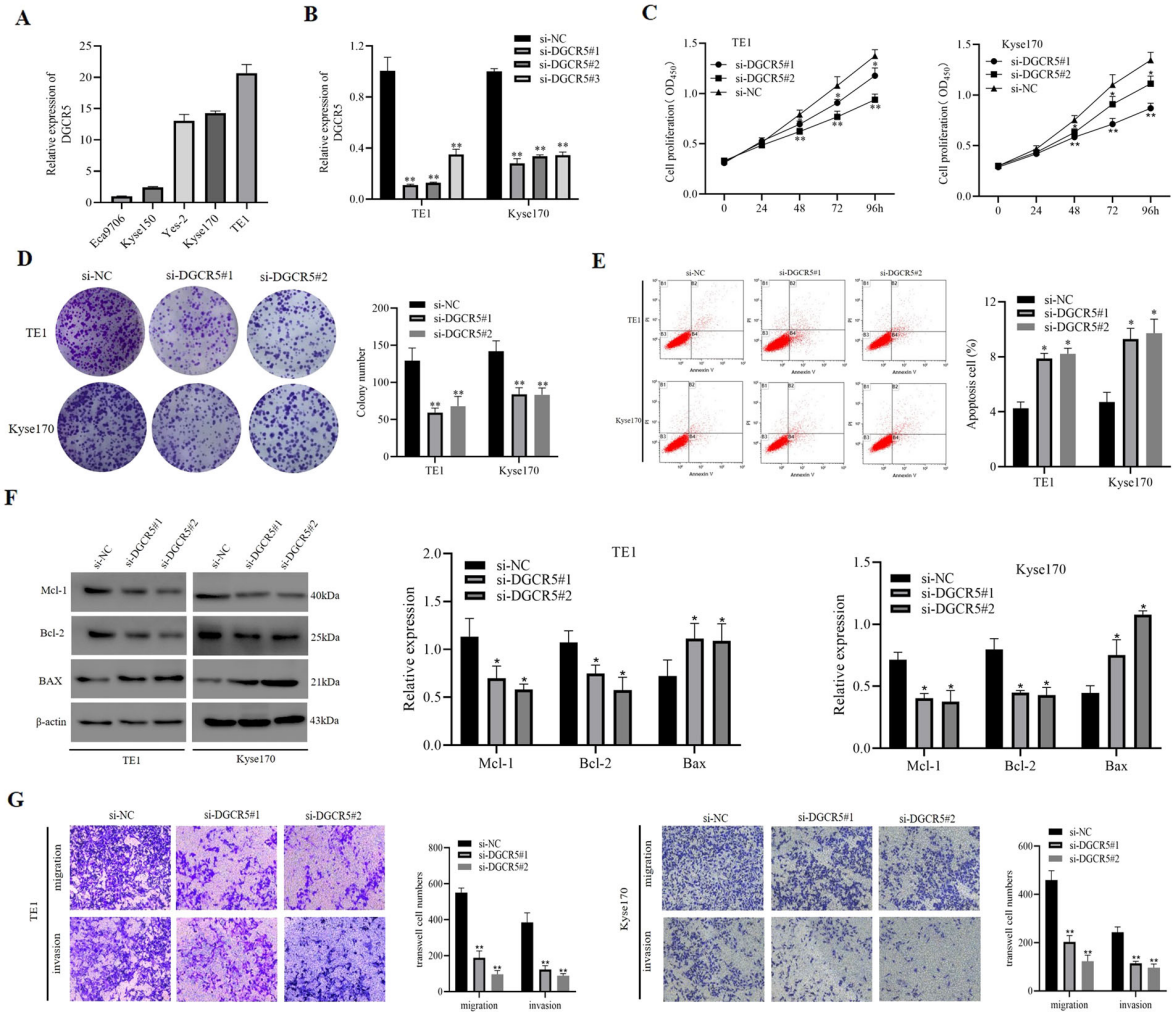
The expression and functions of DGCR5 in TE1 and Kyse170 cells. **A** DGCR5 expression on ESCC cells (Eca9706, TE1, Yes-2, Kyse150, and Kyse170) was examined by qRT-PCR. **B** qRT-PCR analysis for expression levels of DGCR5 on TE1 and Kyse170 cells after transduction with siRNA-DGCR5. **C** Cell proliferation analysis was performed with CCK-8 assays. Cell viabilities were measured at five different time points. **D** DGCR5 knockdown remarkably inhibited cell proliferation by colony formation. **E**, **F** Verification of DGCR5 knockout affects the apoptosis on ESCC cells by FACS and western blot. **G** DGCR5 knockdown inhibited cell migration and invasion by transwell migration and invasion assays. *P < 0.05 and **P < 0.01.

To characterize the function of DGCR5 in ESCC cells, we detected its expression in human ESCC cell lines Eca9706, TE1, Yes-2, Kyse150, and Kyse170 by qRT-PCR. Compared with Eca9706 and Kyse150 cells, DGCR5 expression was higher in TE1, Kyse170, and Yes-2 cell lines (Fig. 2A). To investigate its biological functions, DGCR5 expression in TE1 and Kyse170 cells was successfully knocked down with short interfering RNAs (siRNAs) (Fig. 2B). First, the CCK-8 assay confirmed that silencing DGCR5 significantly suppressed the proliferation of TE1 and Kyse170 cells compared to control groups (Fig. 2C). Colony-formation assays consistently confirmed that the colony-formation ability was inhibited with DGCR5 depletion (Fig. 2D). Second, cell apoptosis analysis by flow cytometry (FCM) performed to that the ESCC cell apoptosis rate in the silencing of the DCGR5 group was significantly increased (Fig. 2E). In addition, western blot examined the effect of DGCCR5 on apoptosis-related proteins and found out the expression of B-cell lymphoma-2 (Bcl-2) and Mcl-1 were both significantly decreased, while Bcl-2-associated X (Bax) was increased after DGCR5 knockdown (Fig. 2F). These data indicated that DGCR5 had a great effect on leading to apoptosis of ESCC cells. Finally, DGCR5 depletion decreased ESCC cell migration and invasion abilities by transwell assays (Fig. 2G). Moreover, the wound-healing assay consistently revealed that knockdown DGCR5 could inhibit cell motility (Supplementary Fig. 2A). Conversely, overexpressing DGCR5 significantly promoted TE1 and Kyse170 cell viability, invasion and migration while inhibited cell apoptosis (Supplementary Figs. 1 and 2B). To sum up, these results displayed that DGCR5 could exert an oncogenic role in the malignant behaviors of ESCC cells.

### DGCR5 physically interacts with SRSF1 protein in ESCC cells

**Fig. 3:**
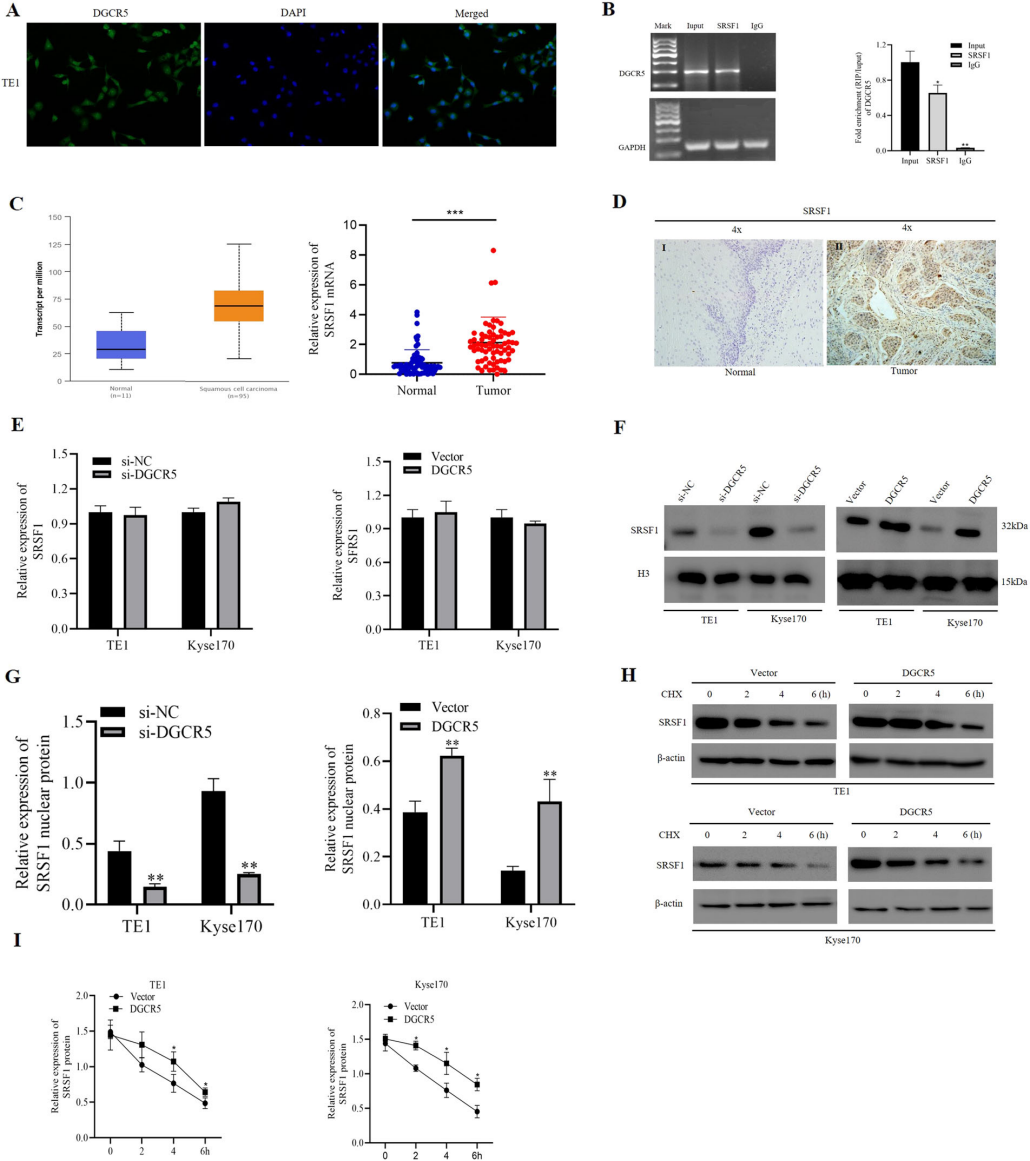
DGCR5 interacts with SRSF1 and regulates the function of SRSF1 on ESCC cells. **A** The subcellular location of DGCR5 in TE1 cells was investigated by FISH. Original magnification ×400. **B** DGCR5 was enriched by SRSF1 in TE1 cell lysates by RIP assays. The expression of DGCR5 was detected by RT-PCR (left) and qRT-PCR (right), which was normalized by GAPDH. **C** SRSF1 mRNA expression was measured in ESCC tissues compared to non-tumor tissues (the former was detected from the TCGA database, the latter was detected from collected 70 clinical specimens by qRT-PCR. **D** IHC staining of SRSF1 in the 20 paired ESCC tissues and non-tumor tissues. Example I showed low SRSF1 staining in ESCC non-tumor tissues. Example II showed high SRSF1 staining in ESCC tissues. **E** qRT-PCR was examined SRSF1 mRNA expression in TE1 and Kyse170 cells transduced with knockdown or overexpression DGCR5. **F**, **G** SRSF1 nucleus protein expression by western blotting in TE1 and Kyse170 cells transduced with knockdown or overexpressed DGCR5. **H** Western blotting analysis of SRSF1 protein in TE1 and Kyse170 cells transduced with overexpressed DGCR5 after 20 µl cycloheximide (CHX) for various times. **I** Triplicated experiments were performed for statistical analysis about SRSF1 protein with overexpressed DGCR5 after CHX in ESCC cells. *P < 0.05 and **P < 0.01.

Then, to elucidate the molecular mechanisms underlying DGCR5 functioning on ESCC cells, we first detected the subcellular location of DGCR5 on ESCC cells by fluorescence in situ hybridization (FISH) and subcellular fractionation. The results showed DGCR5 primarily localized in the nucleus of TE1 and Kyse170 cells (Fig. 3A and Supplementary Fig. 2C, D). Since lncRNAs located in the nucleus can interact with RNA-binding proteins, thus contribute to both RNA processing and protein modifications^25,26^ we conducted the analysis from StarBase v2.0 (http://starbase.sysu.edu.cn/index.php) online database to identify proteins that interacted with DGCR5. As showed SRSF1 mediating the aberrant AS of BIN1 in NSCLC in our previous study, SRSF1 protein caught our attention for its role in splicing regulation in cancers (Supplementary Table 3). We confirmed that SRSF1 protein had significant enrichment of DGCR5 in immunoprecipitated TE1 cells lysate with respect to negative control by RNA-binding protein immunoprecipitation (RIP) assays (Fig. 3B), suggesting there was a direct interaction between DGCR5 and SRSF1 protein in ESCC cells. To further confirm whether DGCR5 could regulate ESCC progression through combing with SRSF1 protein, we analyzed the expression of SRSF1 in ESCC tissues and matched adjacent normal tissues. We found that SRSF1 was significantly upregulated in ESCC tissues (Fig. 3C). In addition, we performed immunohistochemistry (IHC) to show a similar trend, the staining of SRSF1 was mainly found in the nucleus of ESCC carcinoma tissues (Fig. 3D). Among the 20 tumor tissues, 13 cases (65%) showed high expression of SRSF1, whose expression rate was significantly higher than that of ESCC adjacent tissues (*P* < 0.01) (Supplementary Table 4), confirming SRSF1 was considered as a potential cancer-related gene in ESCC.

In order to explore whether oncogenic splicing factor SRSF1 is regulated by DGCR5, we detect SRSF1 expression on ESCC cells when DGCR5 overexpression or knockdown. The consequence showed that there was no correlation between DGCR5 and SRSF1 mRNA level (Fig. 3E). However, knockdown DGCR5 led to a significant downregulation at the protein level of SRSF1, while DGCR5 overexpression increased SRSF1 protein expression, suggesting that DGCR5 could directly regulate SRSF1 expression at protein level (Supplementary Fig. 3A, B). Considering the effect mechanism of DGCR5 on SRSF1 protein, we further analyzed SRSF1 protein expression in the nucleus and cytoplasm of TE1 and Kyse170 cells regulated by DGCR5. Importantly, we found there were no significant changes in cytoplasmic expression of SRSF1 with transfected DGCR5 (Supplementary Fig. 3C, D). However, the expression changes of SRSF1 protein in the nucleus of ESCC cells were observed (Fig. 3F, G), which manifested DGCR5 modulating the oncogenic splicing factor SRSF1 by posttranscriptional regulation in the nucleus of ESCC cells. As lncRNAs showed a potential role to increase the stability of proteins via direct interaction, we performed the protein synthesis inhibitor cycloheximide (CHX) to detect the stability of SRSF1 regulated by DGCR5. We found the stability of the nucleus SRSF1 protein was upregulated in DGCR5 overexpressed ESCC cells (Fig. 3H, I). Based on the above results, we draw a conclusion that DGCR5 could physically interact with SRSF1 protein and act as a posttranslational regulator in ESCC cells.

### DGCR5 upregulates Mcl-1 expression in ESCC cells

**Fig. 4:**
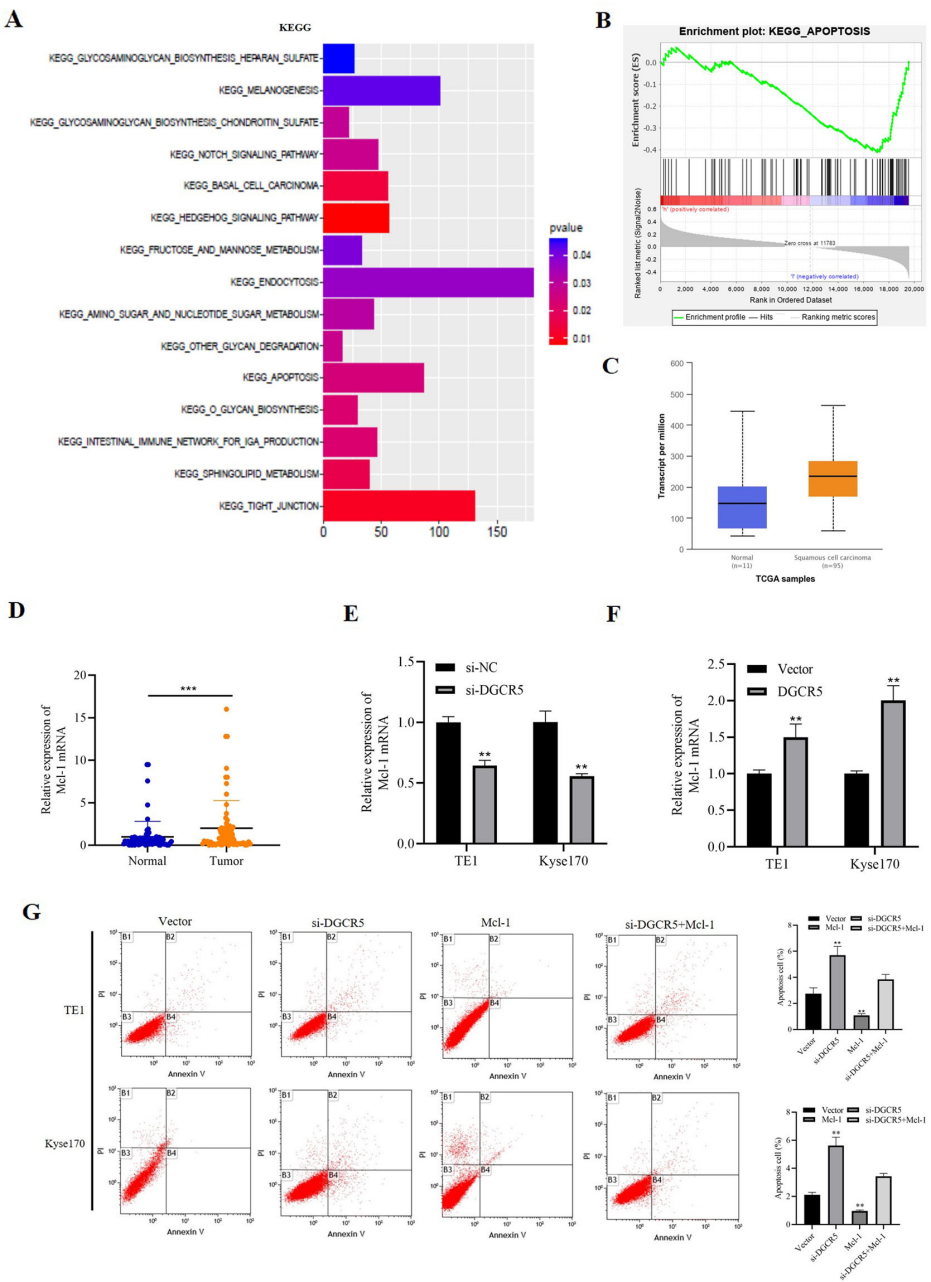
DGCR5 promotes antiapoptotic gene Mcl-1 expression in ESCC. **A** Kyoto Encyclopedia of Genes and Genomes (KEGG) pathway of DGCR5‑related ESCC genes. Fisher exact *P* values were plotted for each enriched functional category. Enriched functional categories with *P* < 0.05 are shown. **B** GESA enrichment of the subsets of DGCR5‑related genes involved in apoptosis. **C** Mcl-1mRNA was upregulated in ESCC tissues according to the TCGA database. **D** Mcl-1 mRNA was increased in the 70 pairs of ESCC samples. **E** Mcl-1 mRNA was inhibited when DGCR5 knockdown in TE1 and Kyse170 cells. **F** Mcl-1 mRNA was upregulated when DGCR5 overexpressed in TE1 and Kyse170 cells. **G** Knockdown of DGCR5 promoted cell apoptosis while Mcl-1 overexpression inhibited apoptosis in TE1 and Kyse170 cells, but simultaneous knockdown DGCR5 and Mcl-1 overexpression could reverse it. *P < 0.05 and **P < 0.01.

To further determine the comprehensive mechanisms of DGCR5 on ESCC progression, we performed the co-expression network analysis of DGCR5-associated genes on ESCC from the TCGA database. A total of 19,567 genes were identified to be DGCR5‑related. Using cBioPortal by setting the Pearson correlation efficient at >0.4, 178 genes were used for the subsequent pathways analysis and gene set enrichment analysis (GSEA). Kyoto encyclopedia of genes and genomes (KEGG) enrichment analysis identified that DGCR5 had a great correlation with the apoptosis signaling pathway (Fig. 4A). Moreover, GSEA showed there was a negative relationship between DGCR5 and the apoptosis signaling pathway (Fig. 4B). CASP8, CSF2RB, PRKACG, Bax, and BCL2L1 genes, involved in GSEA, were highly associated with the expression of DGCR5. Mcl-1, an essential apoptosis-regulatory gene of the Bcl-2 family, was a molecule with efficient clinical significance in various cancers^27^. In addition, considering Mcl-1 has been related to SRSF1^28^, we focused on Mcl-1 as a potential target. Then we confirmed Mcl-1 mRNA expression was significantly upregulated in ESCC samples (Fig. 4C, D). Furthermore, it has been reported that Mcl-1 protein was highly expressed in ESCC tissues, which was a poor prognostic predictor for ESCC patients^29^. To explore whether DGCR5 regulated Mcl-1 expression, we detected the expression of Mcl-1 in DGCR5 knockdown or overexpressed cells. The results showed Mcl-1 expression was obviously inhibited in DGCR5 knockdown cells both at mRNA and protein levels (Figs. 4E and 2F), and vice versa (Fig. 4F and Supplementary Fig. 1F). Then we performed a rescue experiment to define whether DGCR5 inhibited apoptosis of ESCC cells through Mcl-1. We overexpressed Mcl-1 with simultaneous knockdown of DGCR5 and found overexpressed Mcl-1 could partially reverse the increased cell apoptosis caused by knockdown of DGCR5 (Fig. 4G). Therefore, these results demonstrated that DGCR5 inhibited ESCC cell apoptosis by upregulating Mcl-1 expression.

### DGCR5 regulates Mcl-1 mRNA alternative splicing through interacting with SRSF1 protein

**Fig. 5:**
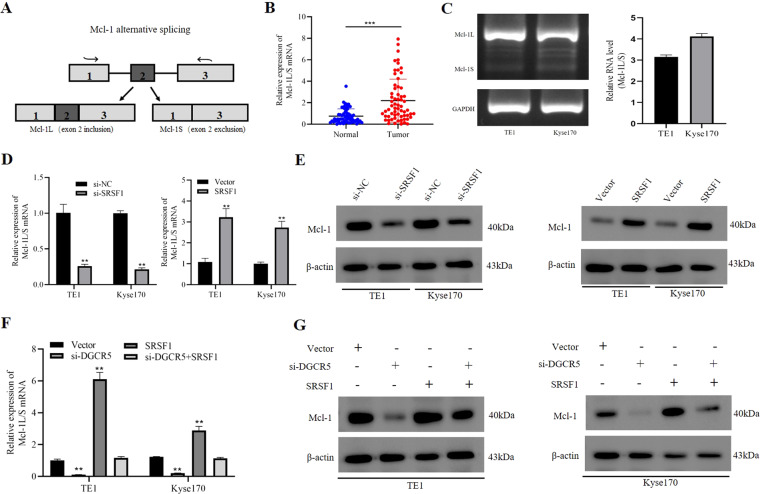
DGCR5 regulates Mcl-1 expression through interacting with SRSF1. **A** SRSF1 regulated the alternative splicing of Mcl-1 pre-mRNA. **B** qRT-PCR was showed the Mcl-1L-to-Mcl-1S ratio in ESCC tissues. **C** Mcl-1L mRNA was obviously increased in TE1 and Kyse170 cells. **D** The Mcl-1L-to-Mcl-1S ratio was detected when SRSF1 knockdown or overexpressed by qRT-PCR. **E** Mcl-1 protein level in ESCC cells was investigated when overexpression or knockdown SRSF1 by western blot. **F**, **G** Knockdown of DGCR5 inhibited the expression of Mcl-1 while SRSF1 overexpression promoted it at the mRNA and protein level, but knockdown DGCR5 and SRSF1 overexpression could reverse it. *P < 0.05 and **P < 0.01.

It is well known that Mcl-1 undergoes alternative splicing events, which generates a short isoform Mcl-1S (pro-apoptotic) and a full-length isoform Mcl-1L (antiapoptotic) (Fig. 5A). We then observed the splicing status of Mcl-1 in ESCC and showed Mcl-1L-to-Mcl-1S ratio was obviously increased in ESCC tissues and cells (Fig. 5B, C). These findings indicated that Mcl-1L isoform was the major form of Mcl-1 high expressed in ESCC. It has reported that SRSF1 mediated the alternative splicing of Mcl-1 mRNA and increased the full-length isoform of antiapoptotic Mcl-1L in cancers^30,31^. To analyze whether the alternative splicing of Mcl-1 regulated by SRSF1 protein in ESCC cells, we successfully constructed SRSF1 overexpressed or knockdown in the TE1 and Kyse170 cells (Supplementary Fig. 4A–F). As the result showed, SRSF1 overexpression could promote a switch in alternative splicing of Mcl-1 toward the antiapoptotic Mcl-1L variant in ESCC cells. In contrast, the opposite results were obtained with SRSF1 knockdown (Fig. 5D and Supplementary Fig. 4G). Furthermore, we examined the expression of Mcl-1 at protein level regulated by SRSF1, which showed overexpression or knockdown SRSF1 could further elevate or reduce the levels of Mcl-1 protein in ESCC cells (Fig. 5E and Supplementary Fig. 5A). These findings indicated that splicing factor SRSF1 was involved in regulating alternative splicing of Mcl-1 in ESCC cells.

To elucidate the effect of DGCR5 on alternative splicing of Mcl-1 via upregulating SRSF1 in ESCC cells, we employed rescue experiments. We found out that the Mcl-1L-to-Mcl-1S ratio of TE1 and Kyse170 cells could be reduced by knocked down DGCR5 but rescued by overexpressing SRSF1, while SRSF1 resulted in the increase in those cells without DGCR5 knockdown (Fig. 5F and Supplementary Fig. 5B). Moreover, DGCR5 knockdown could restrain the expression of Mcl-1 protein in TE1 and Kyse170 cells, while this inhibition could be partially restored by overexpression of SRSF1, and SRSF1 led to a corresponding increase in Mcl-1 protein expression without DGCR5 knockdown (Fig. 5G). These results revealed that DGCR5 could enhance Mcl-1 protein expression of ESCC cells by regulating alternative splicing of Mcl-1 mRNA through interacting with SRSF1 protein. Since the activated caspase-3 pathway is reported to be the activator of central protease in the execution of apoptosis^32^, we then detect the function of the DGCR5/SRSF1/Mcl-1 axis on caspase-3 proteins. The results showed no obvious change was observed in the cleaved-caspase-3 (Supplementary Fig. 5C), indicating that DGCR5 and SRSF1 may regulate apoptosis through a caspase-3-independent pathway in ESCC cells. In a word, these results demonstrated that DGCR5 could involve in alternative splicing of Mcl-1 via regulating SRSF1 in ESCC cells.

### The function of the DGCR5/SRSF1/Mcl-1 axis in vivo

**Fig. 6:**
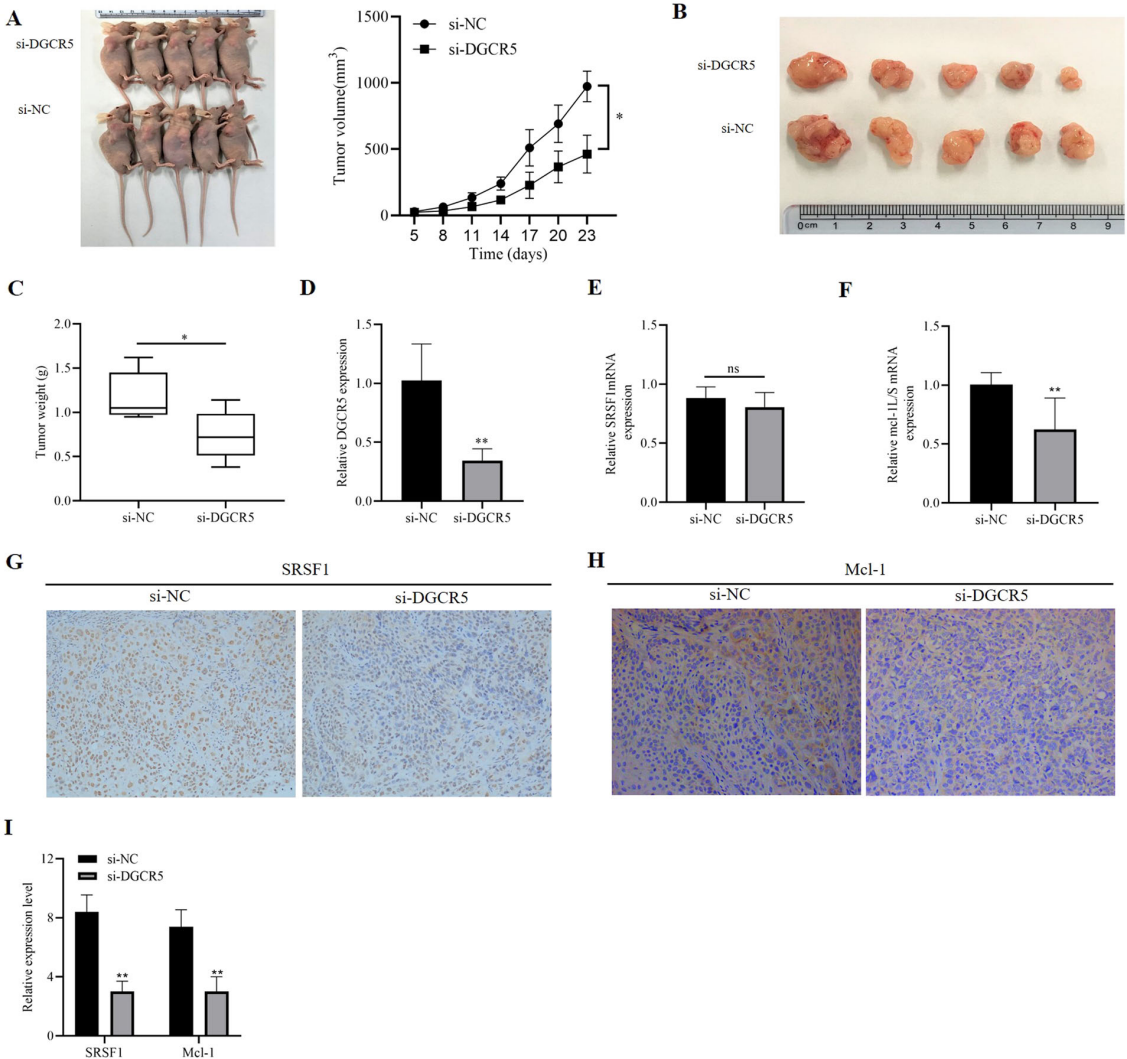
DGCR5 promotes tumor growth in vivo. **A** In total, 4 × 10^6^ Kyse170 cells with si-NC or si-DGCR5 were injected into 5-week-old nude mice subcutaneously. **B**, **C** Weight of subcutaneous xenograft tumors of Kyse170 cells were measured every 3 days, DGCR5 knockdown delayed tumor weight. **D**–**F** Expression level of DGCR5, SRSF1, and Mcl-1 was separately detected by qRT-PCR. **G**–**I** SRSF1 and Mcl-1 expression was measured in tumor tissues by IHC. Original magnification ×200. *P < 0.05 and **P < 0.01.

To further identify the biological role of DGCR5 in tumorigenesis of ESCC in vivo, we established a cell-derived xenograft (CDX) model by subcutaneously injecting Kyse170 cells with vector or interfered DGCR5 into 5-week-old nude mice. Tumor volumes were observed 23 days after injection. We found that tumor volumes and weights in silencing the DGCR5 group were significantly suppressed compared with the control (Fig. 6A–C). DGCR5 expression was significantly knocked down in interfering DGCR5 group (Fig. 6D). These data demonstrated that DGCR5 implied a positive role in tumorigenesis of ESCC cells in vivo. Furthermore, we also confirmed that SRSF1 mRNA expression had no difference between the two groups (Fig. 6E), but the ratio of Mcl-1L/S mRNA significantly decreased in silencing the DGCR5 group (Fig. 6F). In addition, the immunohistochemistry (IHC) staining revealed that ESCC tumor tissues of interfering DGCR5 group had lower expression of SRSF1 and Mcl-1 protein (Fig. 6G–I). In conclusion, these results indicated that DGCR5 could upregulate Mcl-1 expression by its direct interaction with SRSF1 to inhibit apoptosis of ESCC cells, thus leading to attenuation of ESCC cells tumorigenesis in vivo.

## Discussion

**Fig. 7:**
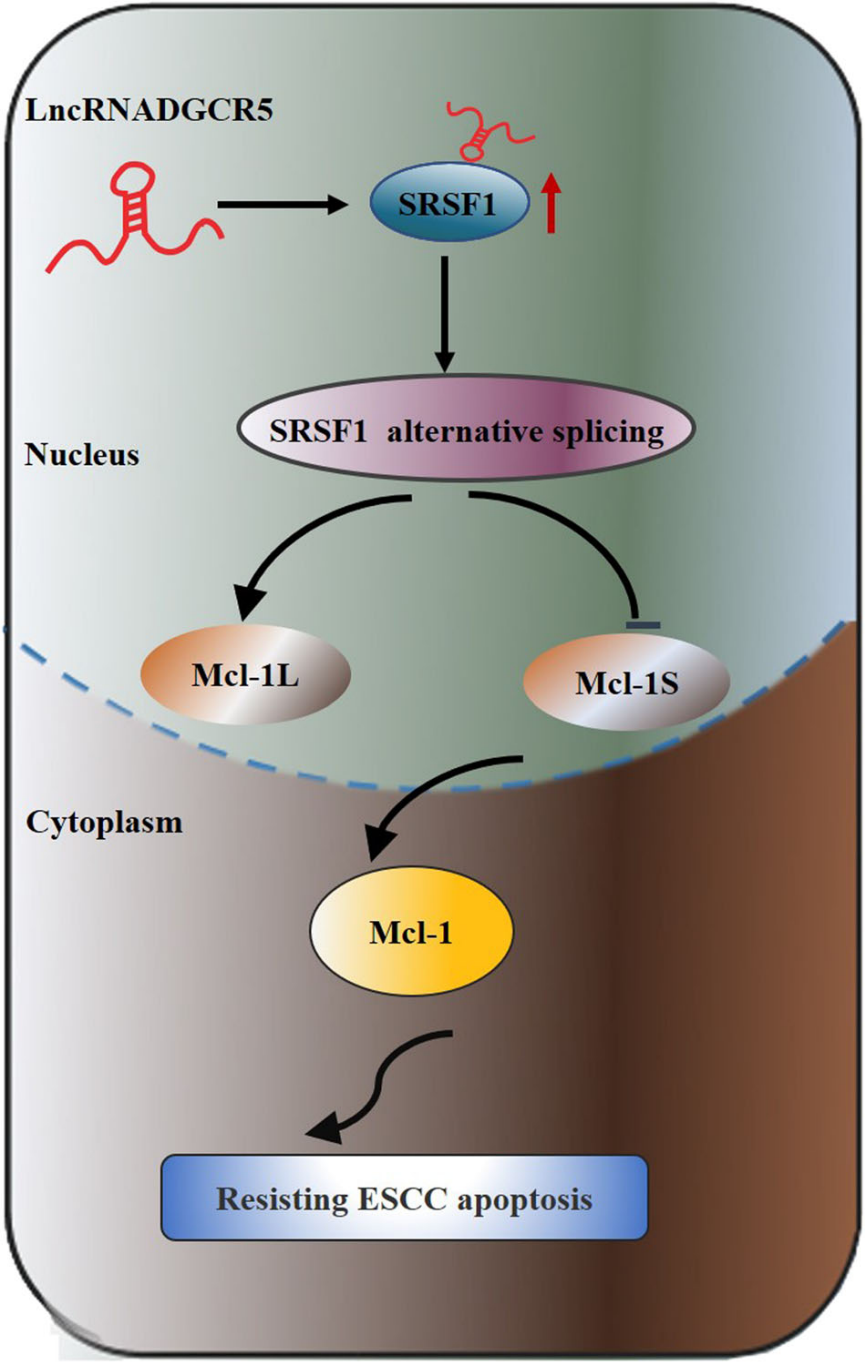
Hypothesis diagram illustrates the mechanism for DGCR5 in ESCC progress. llustrative model showing the mechanism by which DGCR5 involves in tumorigenesisof ESCC via SRSF1-mediated alternative splicing of Mcl-1.

As a highly malignant tumor, the pathogenesis of ESCC remains incompletely understood. Recently, the dysregulation of lncRNAs and their functions in ESCC progression have attracted great attention^11,33^ In this study, we confirmed DGCR5 was highly expressed in ESCC tissues, which was an unfavorable prognosis. Then, we examined that elevated expression of DGCR5 was positively correlated with deeper invasion range, more lymph node metastasis, and higher TNM stage. Knockdown of DGCR5 significantly decreased the proliferation, migration, and invasion ability, while promoted apoptosis of ESCC cells in vitro and in vivo. In addition, we defined the novel mechanism that DGCR5 could connect with the oncogenic splicing factor SRSF1 protein and increase its stability, thus involving the alternative splicing of Mcl-1 mRNA in ESCC cells (Fig. 7). Our findings could provide new insights into the significance of DGCR5 in epigenetic regulation for patients with ESCC.

Increasing evidences show that lncRNAs involve important roles in ESCC, such as proliferation, metastasis, apoptosis, metabolism, and radiotherapy resistance^34–37^, DGCR5 has been identified to participate in the progression in various tumors, such as gallbladder cancer^14^ bladder cancer^19^, non-small cell lung cancer^38^, and glioma^39^. However, the roles of DGCR5 in tumorigenesis of ESCC are not explored. Here, our data discovered that DGCR5 was increased in ESCC tissues comparing with matched adjacent normal tissues. Moreover, elevated DGCR5 expression was significantly associated with higher TNM stage, deep invasion range, more lymph node metastasis. These results proved the basis for the view that high expression of DGCR5 in ESCC tissues could be a new biomarker to indicate tumor prognosis and progression for ESCC patients. Further functional assays were performed to show the biological function of DGCR5, and found that DGCR5 could promote proliferation, invasion, migration, and inhibit apoptosis of ESCC cells. Together, our results characterized the clinical significance of DGCR5 and indicated that DGCR5 might be a potential target for ESCC diagnosis and treatment.

LncRNAs are explored to exert their mechanisms in various biological processes through multiple ways, such as binding with DNA, RNA, or specific proteins, or encoding small peptides^8^. It has been reported nuclear lncRNAs could regulate their biological functions through interacting with proteins to modulate their stability or facilitate the formation of protein complex^40^. Considering DGCR5 dominantly located in the cell nucleus of ESCC, we conducted bioinformatics algorithms to explore potential proteins binding with DGCR5 to determine its possible functional mechanism. Six proteins were found to be potential RBPs interacting with DGCR5, among which SRSF1 caught our attention due to our previous study. Then we confirmed the physical interaction between DGCR5 and SRSF1 protein in ESCC cells by RIP assay. SRSF1, an important oncogene of alternative splicing regulatory, mainly locates in the cell nucleus and correlates with the progression of cancers^41^. Further experiments showed that SRSF1 was also obviously increased in ESCC. Importantly, we identified that DGCR5 was essential for posttranslationally regulating the expression of nuclear SRSF1 protein and increasing its stability in ESCC cells. In brief, these results provided that DGCR5 could interact with SRSF1 protein to regulate its expression in a posttranscriptional way in ESCC cells.

Aberrant AS is demonstrated as a key mechanism that generates multiple RNA variants to regulate protein diversity and enlarge the complexity regulation in many diseases, including cancers^42,43^. SRSF1 protein has been reported to regulate apoptotic control in human cancer cells through alternative splicing pathway^44,45^. Resisting apoptosis is one of the key hallmarks of cancers^46^. As an apoptosis-regulatory gene, Mcl-1 can interfere with mitochondrial events that promoting the release of antiapoptotic factors^47^. In addition, undergoing cancer-relevant AS, Mcl-1 could be produced two functionally distinct variants, Mcl-1S (pro-apoptotic) and Mcl-1L (antiapoptotic), in which the latter variant is predominant in cancers. In particular, Mcl-1 is highly expressed in ESCC and correlates with poor prognosis^48,49^. As previous studies provided, Mcl-1 could be alternatively spliced to exert antiapoptotic functions by SRSF1 protein in breast cancer^50^. In this study, we analyzed the intensive correlation between SRSF1 and Mcl-1 expression in ESCC. Our data revealed that SRSF1 regulated the apoptosis signaling pathway by participating in the alternative splicing of Mcl-1 and triggered the elevation of Mcl-1L subtype in ESCC, providing the novel insight into the role of SRSF1 in Mcl-1 alternative splicing of ESCC cells.

LncRNAs have been confirmed to directly participate in posttranslational regulation of splicing factors in cancers^51^. However, the number of current studies on the correlation of lncRNAs with the regulation of AS events in ESCC is limited. Through bioinformatics analysis, we found that DGCR5 was related to the apoptosis-related signaling pathway. In addition, our research indicated that DGCR5 regulated ESCC cell apoptosis through the Mcl-1 pathway. Then, we explored the influence of SRSF1 on Mcl-1 alternative splicing through DGCR5-mediated in ESCC cells. Our results found that SRSF1 protein was responsible for the regulation of the malignant biological behavior of DGCR5, thereby facilitating the splicing of Mcl-1. These findings demonstrated that the role of the regulatory network between DGCR5/SRSF1/Mcl-1 in ESCC. Moreover, caspase-3 is a downstream target gene of the apoptosis pathway that playing a crucial role in the caspase family^52^. Then we detected the expression of the cleaved-caspase-3 protein in ESCC cells to determine whether it was essential for the DGCR5/SRSF1/Mcl-1axis. Nevertheless, we found that DGCR5 promoted apoptosis of ESCC cells through a caspase-3-independent pathway. Collectively, our findings showed that the interaction between DGCR5 and SRSF1 increased the expression of Mcl-1L via mediating alternative splicing and promoted ESCC cells apoptosis via a caspase-3-independent pathway.

In summary, we determined upregulated expression of DGCR5 in ESCC and verified its great significance for ESCC patients. DGCR5 led to the progression of ESCC cell tumorigenesis both in vitro and in vivo. Moreover, our findings revealed the molecular mechanism that DGCR5 involved in tumorigenesis of ESCC via SRSF1-mediated alternative splicing of Mcl-1. Overall, our study certificated DGCR5/SRSF1/Mcl-1axis could act as a novel regulatory factor in the carcinogenesis of ESCC.

## Materials and methods

### Specimens and cell lines

Seventy pairs of ESCC tissues and matched normal tissues were obtained from radical surgery of ESCC patients in the Fourth Hospital of Hebei Medical University (Shijiazhuang, China) between March 2017 and April 2018. None of the ESCC patients received radiotherapy, chemotherapy, or immunotherapy before surgery. The samples were diagnosed independently by two pathologists. We collected and updated patients’ clinical information every 3 months by follow-up. This study was approved by the ethics committee of the Fourth Hospital of Hebei Medical University.

All human ESCC cell lines (TE1, Eca9706, Kyse170, Kyse150, Yes-2) were obtained from the Research center of the Fourth hospital of Hebei Medical University (Shijiazhuang, China). ESCC cell lines were cultured in RPMI-1640 (Gibco, USA) supplemented with 10% fetal bovine serum (Gibco, USA) in the humidified atmosphere of the 5% CO_2_ incubator. All experiments were performed with mycoplasma-free cells.

### Quantitative RT-PCR (qRT-PCR)

Total RNA was extracted using the Trizol reagent (Invitrogen, USA). PARIS Kit (Invitrogen, USA) was used to isolate the nuclear and cytoplasmic RNA. In total, 2 μg of the total RNA performed to reverse transcription with reverse transcriptase (Thermo Fisher Scientific, USA). The cDNA was subjected to PCR amplification (95 °C for 2 min followed by 35 cycles of 95 °C for 15 s, 58 °C for 15 s, 72 °C for 30 s, and an extension for 10 min at 72 °C) using primers designed for human DGCR5 and Mcl-1, then were separated using 2% agarose gels and visualized with ethidium bromide. SYBR Green Master Mix (ABclonal, China) was used for qRT-PCR in ABI Quant Studio TM 6 Flex. The relative expression of lncRNA and mRNA levels were normalized to GAPDH or small nuclear U6, respectively, and then analyzed by the 2 − ΔCt or 2 − ΔΔCt method. All primers are listed in Supplementary Tables 5 and 6.

### Cell transfection

DGCR5, SRSF1, or Mcl-1 were, respectively, cloned into pCDNA3.1 plasmid for DGCR5, SRSF1, or Mcl-1 overexpression, which were purchased from GenePharma (Suzhou, China). DGCR5 and SRSF1 siRNAs were also synthesized by GenePharma. Transfection was conducted with Lipofectamine 2000 (Invitrogen). Cell transfection was performed as we previously described^10^. The siRNA sequences are as follows Supplementary Table 7.

### CCK-8 assays and colony-formation assays

Transfected cells were seeded into 96-well plates at a density of 1 × 10^3^ cells/well for CCK-8 assays, then was performed as we previously described^10^. For the colony-formation assays, 1 × 10^3^ transfected ESCC cells were inoculated into six-well plates and cultured at 37 °C for 14 days. The colonies were fixed with 4% paraformaldehyde, then stained with 1% crystal violet and counted.

### Migration and invasion assays

For invasion and migration assays, the upper chamber of each insert coated with 20 μl Matrigel to form a matrix barrier or not, 2 × 10^5^ transfected cells suspended in 200 μl of serum-free RPMI-1640 medium were seeded in the upper chamber (Corning, USA), and 600 μl RPMI-1640 medium supplemented with 10% FBS (BI) was placed in the lower chamber. After incubation for 36 h, the cells were fixed with 4% paraformaldehyde for 20 min at room temperature, then stained with 0.1% crystal violet for 20 min. Cells on the lower side of the filters were defined as migration or invasion cells and counted at ×200 magnification in three random fields of each filter. We performed invasion and migration assays as previously described^10^.

### Apoptosis analysis

Apoptosis assays were assayed with flow cytometry (FCM) using the FITC Annexin V Apoptosis Detection Kit I (BD Biosciences, San Jose, USA), which was performed as previously described^10^.

### FISH

TE1 and Kyse170 cells were seeded until cell confluence reached 60–70%, fixed in 4% paraformaldehyde for 20 min, and then added proteinase K (20 μg/ml) to cover cells and incubate for 30 min at 37 °C. Rabbit serum was added for blocking after hybridization with RNA probe (Servicebio, Wuhan, China). Then anti-DIG-HRP was incubated at 37 °C for 40 min. TSA chromogenic reagent added and reacted in the dark for 5 min at room temperature. DAPI was incubated for 8 min in the dark, and then a fluorescence microscope was mounted.

### RNA immunoprecipitation (RIP)

The EZ-Magna RIP kit (Millipore Corp, Billerica, USA) was used for RNA immunoprecipitation (RIP) according to the manufacturer’s instructions. TE1 cells were lysed in RIP buffer consisting of 150 mM KCL, 25 mM Tris-HCl (pH 7.4), 5 mM EDTA, 5 mM DTT, 0.5% Triton X-100, supplemented with RNase inhibitor Ribolock and proteinase inhibitor cocktail. The lysate was mixed with SRSF1 antibody (Santa Cruz Biotechnology, USA) or normal IgG coupled beads and left under rotation at 4 °C. Beads were subsequently washed in lysis buffer and the input RNA was purified, and thus analyzed by RT-PCR and qRT-PCR using the primer of DGCR5. RIP assay was performed as we previously described^10^.

### Western blot analysis

Total proteins were extracted and lysed with RIPA (Biosharp, Hefei, China). Nuclear and cytoplasmic proteins were extracted with the nuclear and cytoplasmic protein extraction kit (Beyotime Biotechnology, Nantong, China). Proteins (30 μg) were separated by 15% SDS-PAGE and transferred onto PVDF membranes (Millipore, Billerica, USA), then blocked with 5% skimmed milk and incubated overnight at 4 °C with the following antibodies: anti-SRSF1 (1:1000; Santa Cruz; sc-73026), Mcl-1 (1:1000; Santa Cruz; sc-74437), Mcl-1 long-form for the molecular weight of 40 kDa and Mcl-1 short-form was 32 kDa. Bcl-2 (1:1000; ABclonal; A0280), BAX (1:1000; ABclonal; A0207), Caspase-3 (1:1000; Proteintech; 19677-1-AP), Histone-H3 (1:1000; Proteintech; 17168-1-AP), or β-actin (1:1000; ABclonal; AC026). Then the species-matched secondary antibodies were incubated for 1 h at 37 °C and the proteins were detected using BeyoECLPlus (Beyotime, China).

### Immunohistochemistry (IHC)

IHC analysis was performed as we previously described^53^. The rabbit polyclonal antibody against human SRSF1 (1:100; Abcam) and the mouse polyclonal antibody against human Mcl-1 (1:100; Santa Cruz) were used for detection. The staining was visualized according to the 0–4 semi-quantitative system, which based on the percentage of positive cells and the intensity of staining. The total scores were determined by multiplying the percentage score and intensity score and considered as a low expression for the score of 0–4 and high expression for the score of 5–12. The immunostaining score was assessed by two pathologists, independently.

### Animal experiment

Four-week-old male BLAB/c nude mice were randomly divided into two groups (*n* = 5/group). In total, 4 × 10^6^ Kyse170 cells with different transfected were suspended in 200 μl serum-free RPMI-1640, then subcutaneously injected into the right flank of each mouse. The tumor was measured every 3 days. The mice were sacrificed after 23 days, then tumors were weighed and processed for further analysis. Tumor volume was calculated as follows: V (volume) = (length × width^2^)/2. All animal experiments were performed under approval by the Fourth Hospital of Hebei Medical University Animal Care Commission.

### Statistical analysis

All statistical analyses were performed using the statistics software, version 20.0 (SPSS, Chicago, IL). The measurement data are presented as mean ± standard error. The Student’s *t* test was employed to compare the differences between the two groups. The correlation between DGCR5 expression and clinicopathological parameters of ESCC patients was detected by chi-square test. For survival analysis, the data were calculated by Kaplan–Meier method and analyzed by log-rank test. *P* < 0.05 was considered to be statistically significant. Graphs were generated by GraphPad Prism 8.

## Supplementary information

Supplementary figure legends

Univariate Cox regression analysis of the relationship between clinicopathological features and survival rate of ESCC patients

Multivariate Cox regression analysis of the relationship between clinicopathological features and survival rate of ESCC patients

List of DGCR5 target proteins by starBase V2.0

Expression of SRSF1 staining between ESCC tissues and matched adjacent tissues n (%)

Real Time Primers

RT-PCR primers

siRNA sequences

DGCR5 overexpression promoted ESCC progression

DGCR5 promotes ESCC cells migration in vitro, and the subcellular location of DGCR5 in ESCC cells.

DGCR5 had no effect on the expression of SRSF1 protein in cytoplasm.

The expression of SRSF1 at mRNA and protein levels were examined by transfection of si-SRSF1 or SRSF1 on TE1 and Kyse170 cells.

DGCR5 promotes the expression of Mcl-1 by activation of SRSF1on TE1 and Kyse170 cells.
