# Supplementary material for: Long noncoding RNA DGCR5 involves in tumorigenesis of esophageal squamous cell carcinoma via SRSF1-mediated alternative splicing of Mcl-1
Source: Cell Death Dis. 2021 Jun 7;12(6):587. doi: 10.1038/s41419-021-03858-7 (PMC8184765; doi:10.1038/s41419-021-03858-7)
Supplement: Supplementary file 1 — Supplementary figure legends [file 41419_2021_3858_MOESM1_ESM.docx]

**Supplementary Fig. 1 DGCR5 overexpression promoted ESCC progression. A** DGCR5 was successfully overexpressed in TE1 and Kyse170 cells by qRT-PCR. **B, C** CCK-8 assay showed that DGCR5 overexpression promoted cell proliferation in TE1 and Kyse170 cells. **D** Colony formation assay detected the effect of DGCR5 overexpression on cell growth ability. **E, F** FCM and Western blot detected that DGCR5 overexpression effects the apoptosis on ESCC cells. **G** Transwell migration and invasion assays revealed that DGCR5 promoted the migration and invasion in both TE1 and Kyse170 cells. *P<0.05 and **P < 0.01.

**Supplementary Fig. 2 DGCR5 promotes ESCC cells migration in vitro, and the subcellular location of DGCR5 in ESCC cells. A, B** Wound healing assays were used to investigate the migratory ability of overexpressing or knockdown DGCR5 on TE1 and Kyse170 cells (200 x). **C** The subcellular location of DGCR5 in Kyse170 cells was investigated by FISH. Original magnification 200 x. **D** The expression of DGCR5 in cytoplasm and nucleus of TE1 and Kyse170 cells was measured by qRT-PCR. GAPDH was a cytoplasmic control, U6 was a nuclear control. *P<0.05 and **P < 0.01.

**Supplementary Fig. 3 DGCR5 had no effect on the expression of SRSF1 protein in cytoplasm. A** The expression of total SRSF1 protein was inhibited by knockdown of DGCR5 by western blot. **B** The expression of total SRSF1 protein was increased by overexpressed of DGCR5 by western blot. **C** The expression of SRSF1 in cytoplasm was no obvious change by knockdown DGCR5 by western blot. **D** The expression of SRSF1 in cytoplasm had no effect on overexpressed DGCR5 by western blot. *P<0.05 and **P < 0.01

**Supplementary Fig. 4 The expression of SRSF1 at mRNA and protein levels were examined by transfection of si-SRSF1 or SRSF1 on TE1 and Kyse170 cells. A** The expression of SRSF1 mRNA was inhibited when knock down SRSF1 by RT-qPCR. **B** The expression of SRSF1 mRNA was increased when overexpression SRSF1 by RT-qPCR. **C, D** The expression of SRSF1 protein expression in TE1 and Kyse170 cells after transfected with si-SRSF1 or SRSF1 detected by western blot. **E, F** The bands gray value of western blot in TE1 and Kyse170 cells after transfected with si-SRSF1 or SRSF1 was examined. **G** The expression of Mcl-1 isoforms was detected in TE1 and Kyse170 cells after knockdown SRSF1 by PCR. *P<0.05 and **P < 0.01.

**Supplementary Fig. 5 DGCR5 promotes the expression of Mcl-1 by activation of SRSF1on TE1 and Kyse170 cells.** **A** The bands gray value of western blot in TE1 and Kyse170 cells after knock down or overexpression SRSF1 was examined. **B** The bands gray value of western blot in TE1 and Kyse170 cells was knocked down by DGCR5 depletion while SRSF1 overexpression promoted it, but knockdown DGCR5 and SRSF1overexpression could reversed it. **C** There was no effect on Caspase3 and cleaved- caspase3 protein in ESCC cells regulated by DGCR5 through with SRSF1. *P<0.05 and **P < 0.01.
