# Supplementary material for: Long noncoding RNA DGCR5 involves in tumorigenesis of esophageal squamous cell carcinoma via SRSF1-mediated alternative splicing of Mcl-1
Source: Cell Death Dis. 2021 Jun 7;12(6):587. doi: 10.1038/s41419-021-03858-7 (PMC8184765; doi:10.1038/s41419-021-03858-7)
Supplement: Supplementary file 2 — Univariate Cox regression analysis of the relationship between clinicopathological features and survival rate of ESCC patients [file 41419_2021_3858_MOESM2_ESM.docx]

Supplementary Table. 1 Univariate Cox regression analysis of the relationship between clinicopathological features and survival rate of ESCC patients

| Parameter | Univariate analysis | | |
| --- | --- | --- | --- |
|  | HR | 95%CI | *P* value |
| Gender | 1.295 | 0.685-2.45 | 0.426 |
| Age | 1.060 | 0.589-1.906 | 0.846 |
| Invasion range | 0.859 | 0.478-1.545 | 0.613 |
| TNM stage | 0.548 | 0.304-0.988 | 0.045 |
| Metastasis (lymph) | 0.531 | 0.284-0.992 | 0.047 |
| DGCR5 level | 2.506 | 1.357-4.629 | 0.003 |
