# Supplementary material for: Long noncoding RNA DGCR5 involves in tumorigenesis of esophageal squamous cell carcinoma via SRSF1-mediated alternative splicing of Mcl-1
Source: Cell Death Dis. 2021 Jun 7;12(6):587. doi: 10.1038/s41419-021-03858-7 (PMC8184765; doi:10.1038/s41419-021-03858-7)
Supplement: Supplementary file 3 — Multivariate Cox regression analysis of the relationship between clinicopathological features and survival rate of ESCC patients [file 41419_2021_3858_MOESM3_ESM.docx]

Supplementary Table. 2 Multivariate Cox regression analysis of the relationship between clinicopathological features and survival rate of ESCC patients

| Parameter | Multivariate analysis | | |
| --- | --- | --- | --- |
|  | HR | 95%CI | *P* value |
| Gender | 1.069 | 0.532-2.15 | 0.851 |
| Age | 1.085 | 0.587-2.005 | 0.795 |
| Invasion range | 1.4 | 0.738-2.656 | 0.303 |
| TNM stage | 1.955 | 0.722-5.292 | 1.87 |
| Metastasis (lymph) | 0.902 | 0.327-2.485 | 0.842 |
| DGCR5 level | 2.478 | 1.28-4.796 | 0.007 |
