# Supplementary material for: Long noncoding RNA DGCR5 involves in tumorigenesis of esophageal squamous cell carcinoma via SRSF1-mediated alternative splicing of Mcl-1
Source: Cell Death Dis. 2021 Jun 7;12(6):587. doi: 10.1038/s41419-021-03858-7 (PMC8184765; doi:10.1038/s41419-021-03858-7)
Supplement: Supplementary file 4 — List of DGCR5 target proteins by starBase V2.0 [file 41419_2021_3858_MOESM4_ESM.docx]

Supplementary Table. 3 List of DGCR5 target proteins by starBase V2.0

| Gen name | Target protein |
| --- | --- |
| DGCR5 | eIF4AIII |
| DGCR5 | FMRP |
| DGCR5 | FUS |
| DGCR5 | LIN28A |
| DGCR5 | SRSF1 |
| DGCR5 | UPF1 |
