# Supplementary material for: Long noncoding RNA DGCR5 involves in tumorigenesis of esophageal squamous cell carcinoma via SRSF1-mediated alternative splicing of Mcl-1
Source: Cell Death Dis. 2021 Jun 7;12(6):587. doi: 10.1038/s41419-021-03858-7 (PMC8184765; doi:10.1038/s41419-021-03858-7)
Supplement: Supplementary file 5 — Expression of SRSF1 staining between ESCC tissues and matched adjacent tissues n (%) [file 41419_2021_3858_MOESM5_ESM.docx]

Supplementary Table. 4 Expression of SRSF1 staining between ESCC tissues and matched adjacent tissues *n* (%)

| SRSF1 expression | ESCC tissues  (*n*=20) | Matched adjacent tissues  (*n*=20) | χ^2^ | *P* value |
| --- | --- | --- | --- | --- |
| Low | 7(35) | 16(80) | 8.286 | 0.004 |
| High | 13(65) | 4(20) |  |  |
