# Supplementary material for: Long noncoding RNA DGCR5 involves in tumorigenesis of esophageal squamous cell carcinoma via SRSF1-mediated alternative splicing of Mcl-1
Source: Cell Death Dis. 2021 Jun 7;12(6):587. doi: 10.1038/s41419-021-03858-7 (PMC8184765; doi:10.1038/s41419-021-03858-7)
Supplement: Supplementary file 6 — Real Time Primers [file 41419_2021_3858_MOESM6_ESM.docx]

**Supplementary Table.5 Real Time Primers**

| Gene | Primer |
| --- | --- |
| GAPDH | F: 5’-GGTGAAGGTCGGAGTCAACG-3’ |
|  | R: 5’-CAAAGTTGTCATGGATGHACC-3’ |
| DGCR5  SRSF1  Mcl-1  Mcl-1L  Mcl-1S | F: 5’-TCAGAGTGGCGAAGGCAGGAG-3’ |
|  | R: 5’-GCAACTCAAGTAAGTCCGCACCT-3’  F: 5’-AGGAGGATTGAGGAGGATCAG-3’  R: 5’-CGCTCCATGAATCCTGGTAA-3’  F: 5’-GAAACGCGGTAATCGGACTC-3’  R: 5’-GCTACTGGCCACTTTCCTGT-3’  F: 5’-GCATCGAACCATTAGCAGAA-3’  R: 5’-AAGCCAGCAGCACATTCCT-3’  F: 5’-GGCCTTCCAAGGATGGGTT-3’  S: 5’-GCTCCTACTCCAGCAACACC-3’ |
