# Supplementary material for: Long noncoding RNA DGCR5 involves in tumorigenesis of esophageal squamous cell carcinoma via SRSF1-mediated alternative splicing of Mcl-1
Source: Cell Death Dis. 2021 Jun 7;12(6):587. doi: 10.1038/s41419-021-03858-7 (PMC8184765; doi:10.1038/s41419-021-03858-7)
Supplement: Supplementary file 7 — RT-PCR primers [file 41419_2021_3858_MOESM7_ESM.docx]

**Supplementary Table.6 RT-PCR primers**

| Gene Primer |
| --- |
| Mcl-1（alternatively spliced） F: 5′- GGACACA AAGCCAATGGGCAGGT-3′  R: 5′-GCAAAA GCCAGCAGCACATTCCTGA-3′ |
