# Supplementary material for: Long noncoding RNA DGCR5 involves in tumorigenesis of esophageal squamous cell carcinoma via SRSF1-mediated alternative splicing of Mcl-1
Source: Cell Death Dis. 2021 Jun 7;12(6):587. doi: 10.1038/s41419-021-03858-7 (PMC8184765; doi:10.1038/s41419-021-03858-7)
Supplement: Supplementary file 8 — siRNA sequences [file 41419_2021_3858_MOESM8_ESM.docx]

**Supplementary Table.7** **siRNA sequences**

| Gene | siRNA sequences（5’-3’） |
| --- | --- |
| si-DGCR5#1  si-DGCR5#2  si-DGCR5#3 | GCAAUUAGCUUCAGCUCUATT |
|  | CCAGUAGUGUUUGUGCUUUTT  GCACAAAGAGAUCCCUCAATT |
| si-SRSF1#1  si-SRSF1#2  si-SRSF1#3 | GCUAUGAUUACGAUGGGUATT |
|  | CUACGUGGGUAACUUACCUTT  GAAAGAAGAUAUGACCUAUTT |
