# Supplementary figures and images for: Long noncoding RNA DGCR5 involves in tumorigenesis of esophageal squamous cell carcinoma via SRSF1-mediated alternative splicing of Mcl-1
Source: Cell Death Dis. 2021 Jun 7;12(6):587. doi: 10.1038/s41419-021-03858-7 (PMC8184765; doi:10.1038/s41419-021-03858-7)

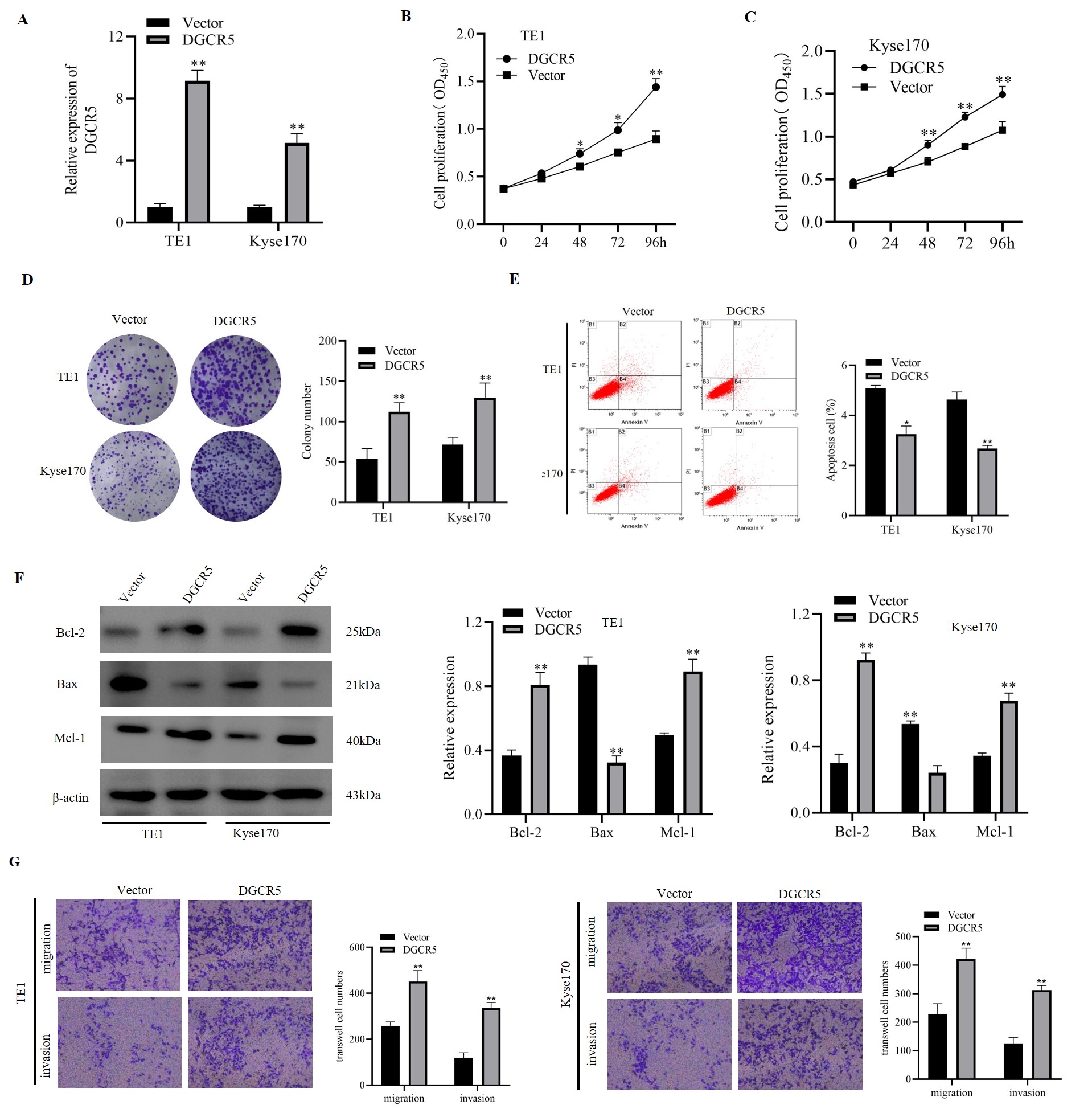

Supplement: Supplementary file 9 — DGCR5 overexpression promoted ESCC progression [file 41419_2021_3858_MOESM9_ESM.jpg]

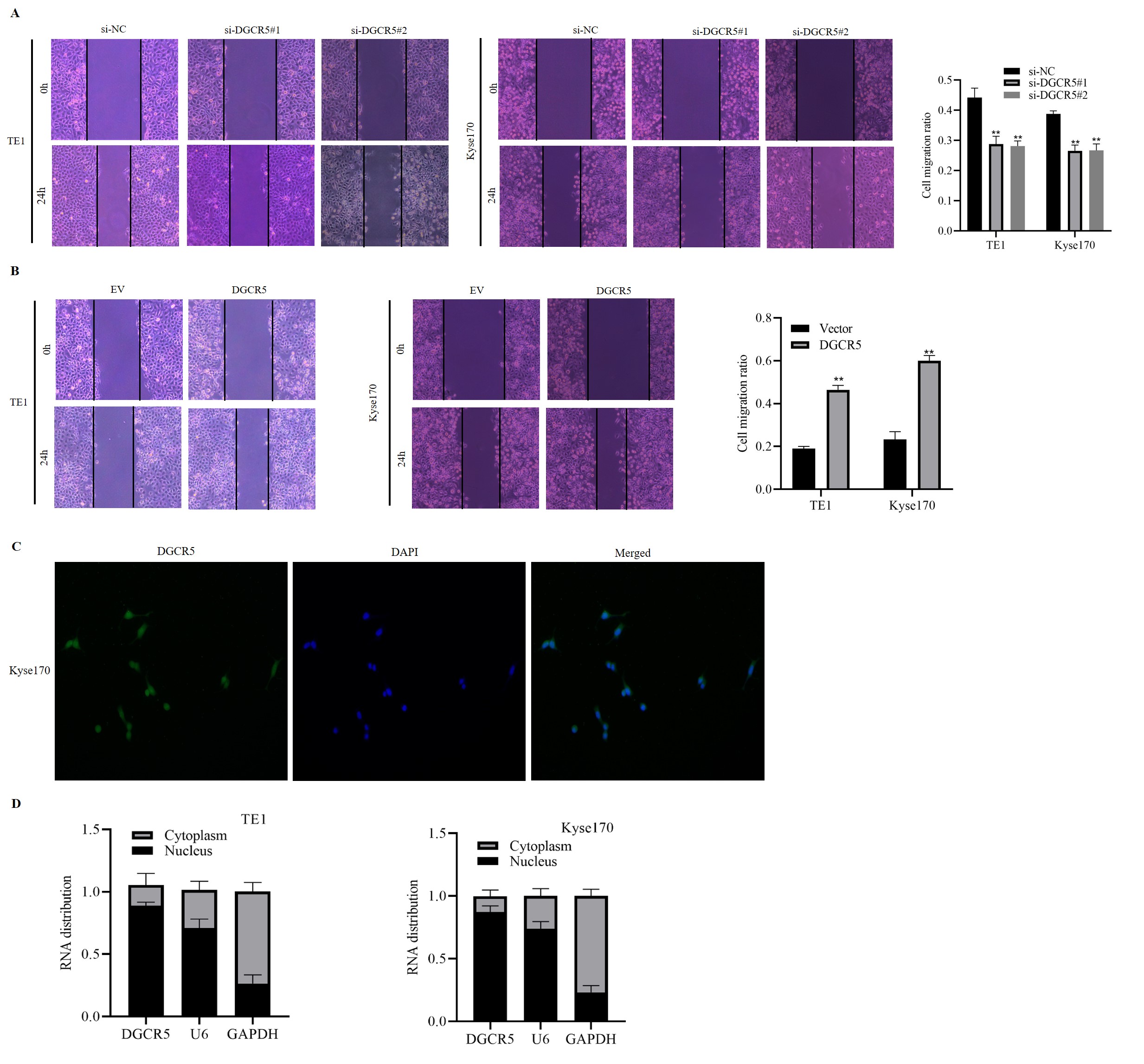

Supplement: Supplementary file 10 — DGCR5 promotes ESCC cells migration in vitro, and the subcellular location of DGCR5 in ESCC cells. [file 41419_2021_3858_MOESM10_ESM.jpg]

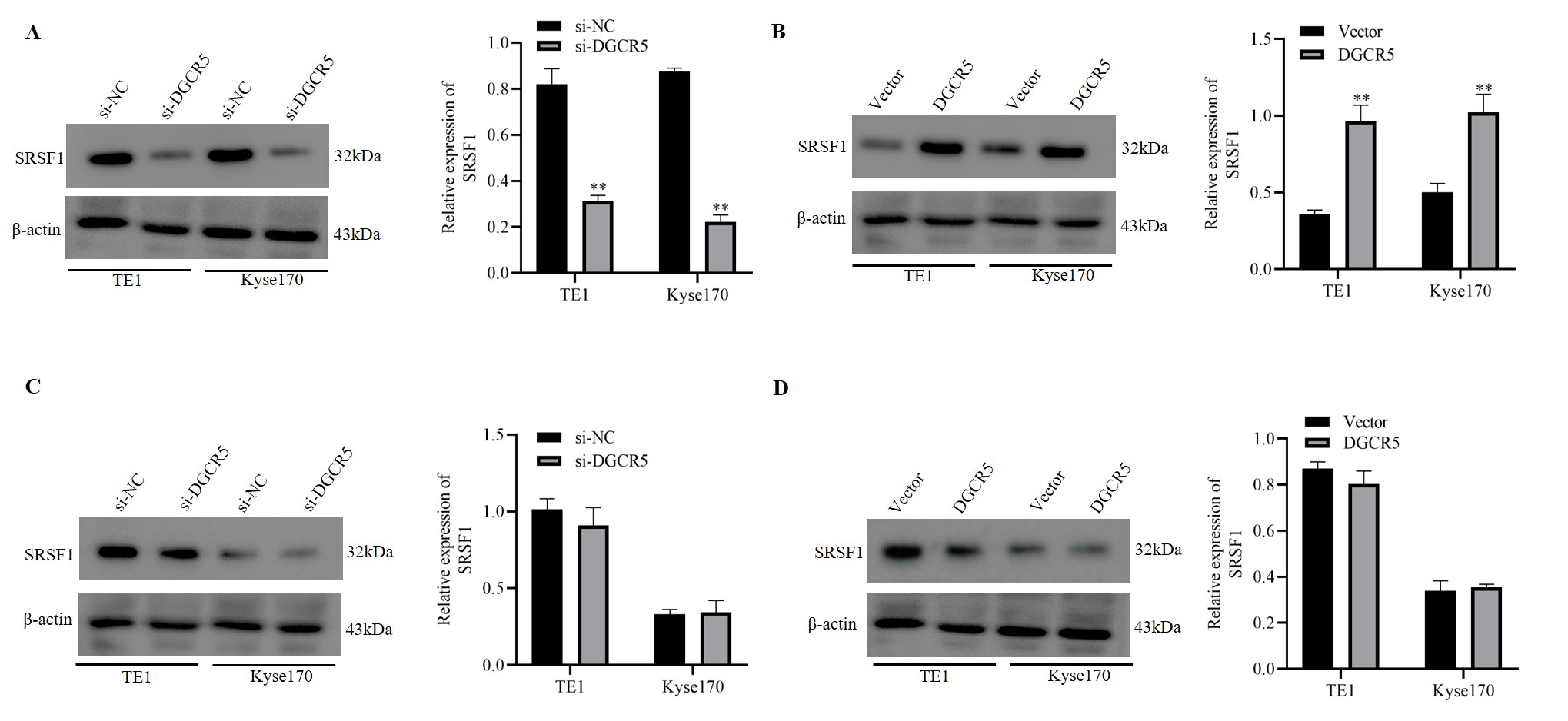

Supplement: Supplementary file 11 — DGCR5 had no effect on the expression of SRSF1 protein in cytoplasm. [file 41419_2021_3858_MOESM11_ESM.jpg]

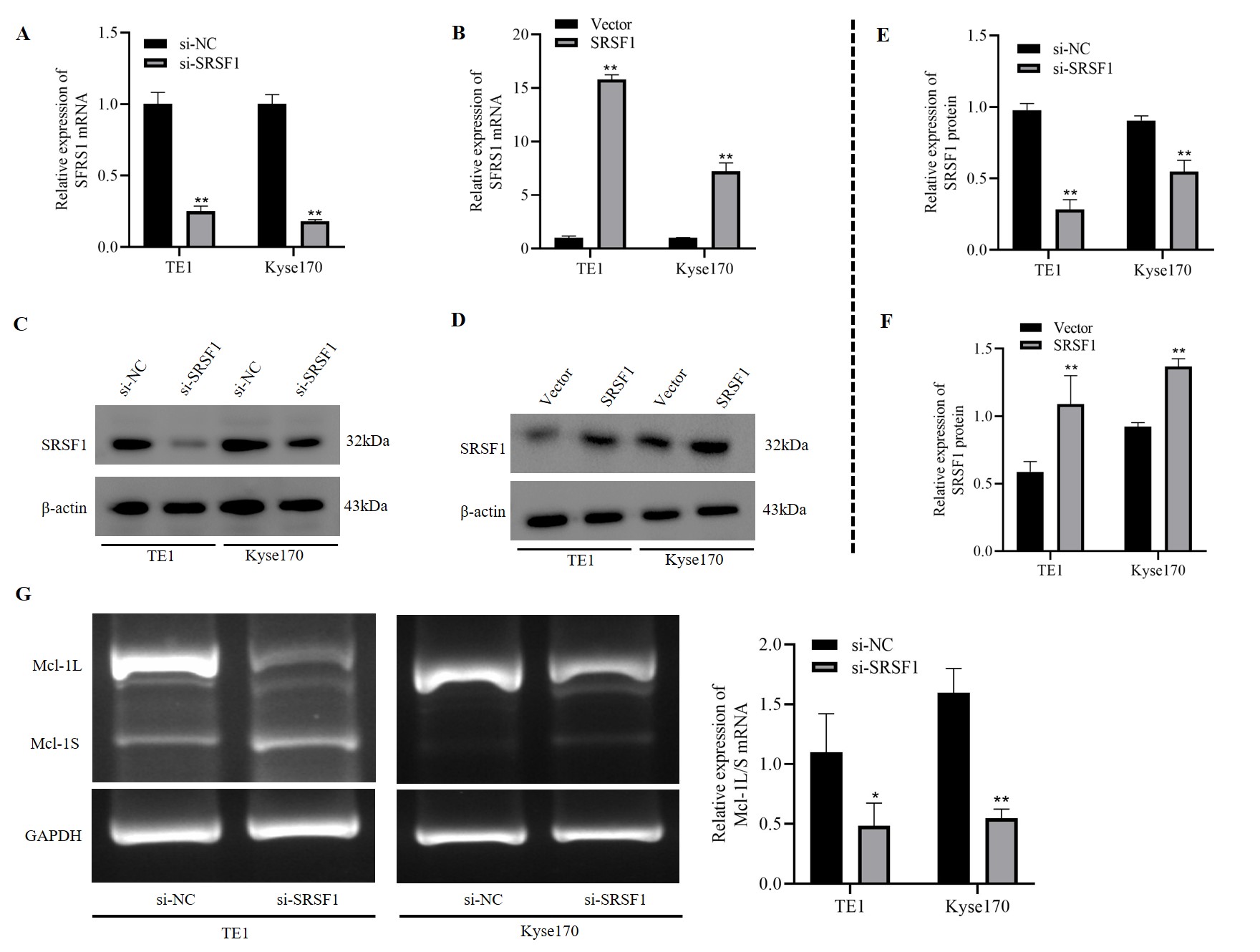

Supplement: Supplementary file 12 — The expression of SRSF1 at mRNA and protein levels were examined by transfection of si-SRSF1 or SRSF1 on TE1 and Kyse170 cells. [file 41419_2021_3858_MOESM12_ESM.jpg]

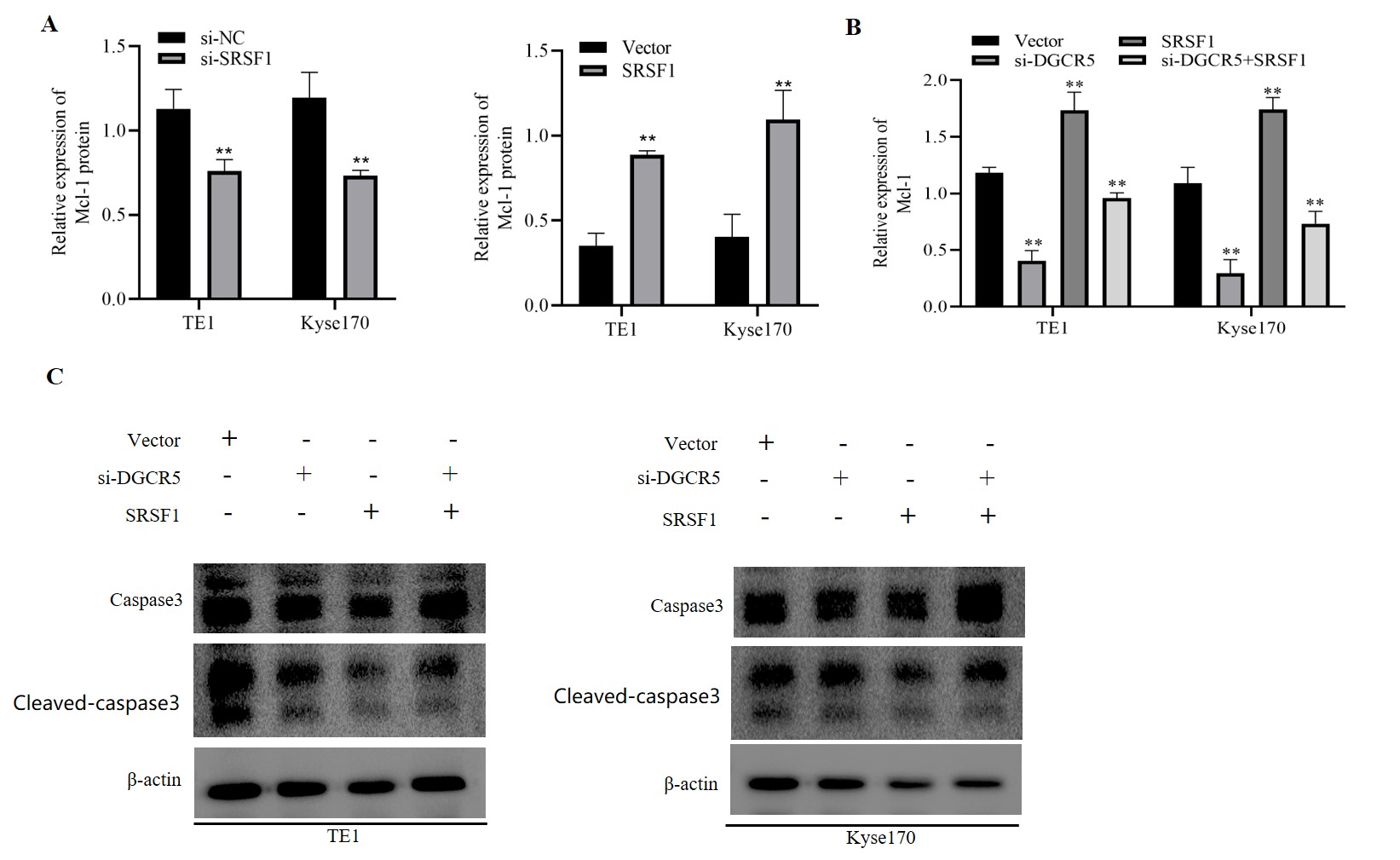

Supplement: Supplementary file 13 — DGCR5 promotes the expression of Mcl-1 by activation of SRSF1on TE1 and Kyse170 cells. [file 41419_2021_3858_MOESM13_ESM.jpg]
